# Laser-Induced Silver Nanoparticles on Titanium Oxide for Photocatalytic Degradation of Methylene Blue

**DOI:** 10.3390/ijms10114707

**Published:** 2009-10-29

**Authors:** Thou-Jen Whang, Hsien-Yu Huang, Mu-Tao Hsieh, Jyun-Jen Chen

**Affiliations:** Department of Chemistry, National Cheng Kung University, No. 1, University Road, Tainan 70101, Taiwan; E-Mails: ryo8888.tw@yahoo.com.tw (H.Y.H.); z8408073@email.ncku.edu.tw (M.T.H.); seed_01@yahoo.com.tw (J.J.C.)

**Keywords:** titanium oxide, photocatalysis, silver nanoparticles, methylene blue, laser

## Abstract

Silver nanoparticles doped on titanium oxide (TiO_2_) were produced by laser-liquid interaction of silver nitrate (AgNO_3_) in isopropanol. Characteristics of Ag/TiO_2_ (Ag doped TiO_2_) nanoparticles produced by the methods presented in this article were investigated by XRD, TEM, SEM, EDX, and UV-Vis. From the UV-Vis measurements, the absorption of visible light of the Ag/TiO_2_ photocatalysts was improved (additional absorption at longer wavelength in visible light region) obviously. The photocatalytic efficiency of Ag/TiO_2_ was tested by the degradation of methylene blue (MB) in aqueous solution. A maximum of 82.3% MB degradation is achieved by 2.0 wt% Ag/TiO_2_ photocatalyst under 2 h illumination with a halogen lamp.

## 1. Introduction

Waste water from the textile industry constitutes a serious environmental problem. Most of the dyestuffs used are difficult to decompose, due to their chemical structures [1]. As the public demand for environmental protection increases and the governmental authorities are more concerned with the enforcement of the corresponding regulations, the top priority is to find an efficient solution dealing with this issue for the time being.

Semiconductor photocatalysts have been a potential candidates for treating various water pollutants [2–4]. After some 30 years of extensive research, many oxide compounds of semiconductor photocatalysts include TiO_2_, ZnO, WO_3_, SnO_2_, and ZrO_2_, and some of the sulfide compounds like CdS, ZnS are among those most interesting materials in this respect [5]. Within these compounds, titanium oxide has also been investigated for its photocatalytic activities [6,7] and for use in photoelectrochemical cells as well [8]. It has drawn great attention in research and industrial fields in recent years because of its characteristics of powerful oxidation capability, non-toxicity, chemical stability, and cost-effectiveness. Nevertheless, one of the drawbacks of TiO_2_ for photocatalytic process is its relative big band gap (3.0 eV for rutile phase and 3.2 eV for anatase phase, respectively). As a result, TiO_2_ absorbs light wavelength less than 388 nm and the photocatalytic processes only occur in this region. Therefore, many studies have employed modifications of TiO_2_ in order to improve its catalysis efficiency through enhancements of its absorbance in the visible light region to match the solar spectrum. Those methods consist of doping with metals [9–11], non-metal ions [12–14], dye-sensitization [15,16] and so forth.

Kondo and Jardim [17] were among the pioneers who incorporated silver into a TiO_2_ matrix for photocatalytical applications. The doping of silver nanoparticles into a TiO_2_ matrix can be achieved by chemical reduction [18], the reverse micellar route [19], irradiation of silver ions in solution [20], and the sol-gel method [21]. Silver doped semiconductor substrate has been studied to enhance the photocatalytical efficiency by trapping the photo-induced charge carriers, especially electrons, and facilitating the transfer process.

In this article, we propose a method of doping Ag nanoparticles onto a TiO_2_ matrix by introducing a laser to the deposition processes, namely via a laser-liquid interaction of AgNO_3_ and TiO_2_ in a liquid medium (isopropanol). In the laser-liquid interaction, nucleation and growth of Ag nanoparticles take place as the amount of Ag atoms being generated in the liquid reaches the condition of supersaturation [22]. The nanoparticles produced by laser-liquid interaction have the advantages of stability in liquid media and narrow diameter distributions. For practical application of this Ag doped TiO_2_ photocatalyst, the efficiency of Ag/TiO_2_ was tested by the degradation of MB in aqueous solution.

## 2. Results and Discussion

### 2.1. Laser-Induced Interaction

Silver nanoparticles deposited on titanium oxide were produced by laser-liquid interaction of TiO_2_ powder and AgNO_3_ dissolved in isopropanol. Different weight ratios of TiO_2_ and AgNO_3_ were sonicated in isopropanol before being irradiated by the focused output of second harmonic (532 nm) of Nd:YAG laser operating at 10 Hz with the power of 25 mJ. Isopropanol was used as the reaction medium to prevent the aggregation of silver nanoparticles while the reaction proceeds. The sizes of the Ag nanoparticles produced by the laser-liquid interaction were found to be about 18~22 nm in diameter. Since the size, shape, and microstructure of Ag particles are dependent on some important parameters, such as power, frequency, and interaction time, those factors were considered in the laser-induced processes.

### 2.2. Characterization of Ag/TiO_2_

**Figure 1.**
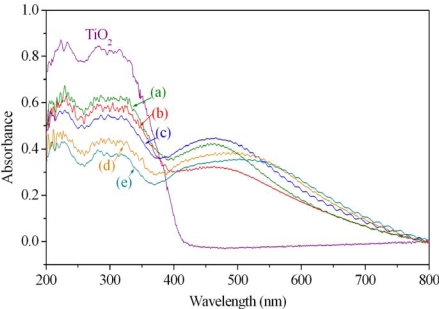
UV-Vis spectra of TiO_2_ and (a) 0.5, (b) 1.0, (c) 2.0, (d) 5.0, and (e) 10.0 wt% Ag in TiO_2_.

The UV-Vis measurements (200~800 nm; 300 nm/min.) of Ag/TiO_2_ photocatalysts are shown in Figure 1. From the results, the absorption curve of TiO_2_ indicates that TiO_2_-only has no absorption in the spectral region above 420 nm. After the doping of Ag nanoparticles onto TiO_2_, the absorption curves of (a)~(e) were obviously improved in the 380~780 nm spectral region. This additional absorption peak in the visible region occurs because of the surface plasmon resonance (SPR), the interference of electromagnetic field with the conduction electrons of silver particles dispersed on the TiO_2_ matrix. The enhanced absorption is indicative of the greater probability of enhancing the photocatalytic efficiency of Ag/TiO_2_ by broadening the light absorption in the visible region with Ag nanoparticles.

It has been reported that the doped metallic nanoparticles on TiO_2_ are acting like electron traps [23], retarding the recombination of electron-hole pairs which were provoked by the photon absorption of the TiO_2_ matrix. We found that among the various ratios of Ag/TiO_2_ produced by laser-liquid interaction, the 2.0 wt% Ag/TiO_2_ [Figure 1 curve (c)] presents the highest photon absorption in the visible light region with λ_max_ (maximum absorption wavelength) located at around 470 nm. Interestingly, the absorption enhancement of Ag/TiO_2_ at visible light region does not correspond to the ratios of silver in the photocatalysts. Although silver nanoparticles help improve the visible light absorption, it is likely that some particles shield the interaction of light as more Ag is deposited on the TiO_2_ matrix. It is also noted that a shifting of the maximum absorptions in the visible light region occurred for various ratios of Ag/TiO_2_, just as curves (d) and (e) in Figure 1 are red-shifted to 500~520 nm, which is attributed to the size differences of the deposited Ag nanoparticles.

**Figure 2.**
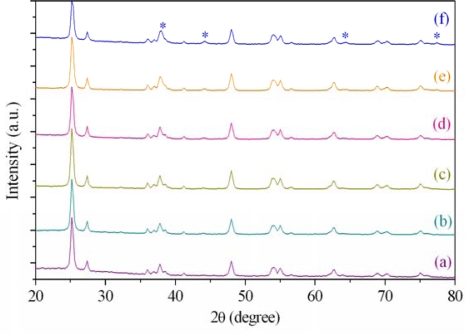
XRD patterns of (a) 0.0, (b) 0.5, (c) 1.0, (d) 2.0, (e) 5.0, and (f) 10.0 wt% Ag in TiO_2_. Ag signals were indicated by the asterisks.

Figure 2 shows the XRD patterns of TiO_2_-only and various ratios of Ag/TiO_2_ produced by the laser-liquid interaction. Silver signals were clearly observed at 38°, 44.2°, 64.6° and 77.2° (2θ) of the patterns among other signals from TiO_2_. As the weight percentages of Ag increase, the signals of deposited Ag are more distinct correspondingly. Compare the peaks of TiO_2_ in Figure 2(a) with those in (b)~(f) from various Ag/TiO_2_, these peaks looked identical to each other. It suggests that a large part of Ag particles were not incorporated in TiO_2_ lattice, but deposited on the surface of the matrix instead. The crystallite size of nanoparticles can be calculated by applying to the Scherrer’s equation:

(1) D = 0.9λ/(Bcosθ)

where *D* is the average crystallite size, 0.9 is the shape factor of the grain, λ is the wavelength of X-ray which is 0.154051 nm for Cu Kα radiation, *B* is the FWHM of the diffraction peak, and θ is the incident angle of X-ray. By the diffraction data in Figure 2, the primary particle size can be measured according to the Scherrer analysis for diffraction peak width at 25.2° (2θ). The estimated sizes are about 17.7 nm, 18.3 nm, 19.0 nm, 19.0 nm, 17.7 nm, and 17.7 nm for TiO_2_-only, 0.5 wt%, 1.0 wt%, 2.0 wt%, 5.0 wt%, and 10.0 wt% Ag/TiO_2_, respectively. The crystallinity of photocatalysts can be estimated by the relative peak areas of XRD to standard of P25 TiO_2_, which is considered to be 100% crystalline, because the intensity of the X-ray scattering is proportional to the peak area above the background scattering [24]. The degree of crystallinity for these photocatalysts are 86.3%, 101.0%, 88.8%, 97.8%, and 83.8% for 0.5 wt%, 1.0 wt%, 2.0 wt%, 5.0 wt%, and 10.0 wt% Ag/TiO_2_, respectively. The results suggest that the use of various amount of silver doping leads to photocatalysts with different crystallinities.

**Figure 3.**
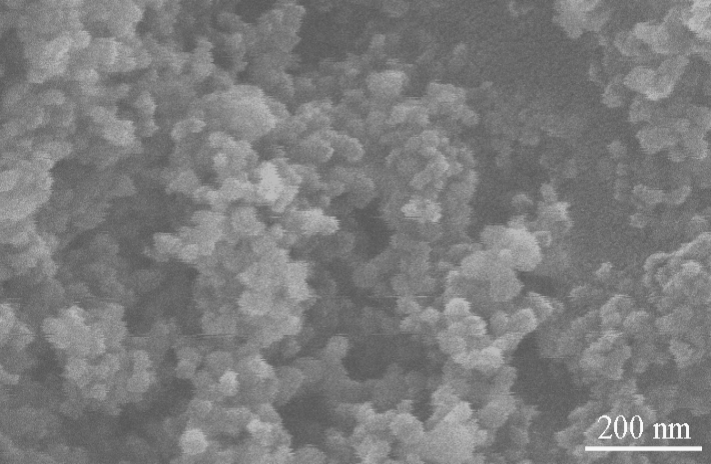
SEM image of 2.0 wt% Ag in TiO_2_ nanoparticles.

The SEM image of 2.0 wt% Ag/TiO_2_ nanoparticles is shown in Figure 3. Some porous surface dispersed among cauliflower-like clusters of grains was observed. The scope of particle size was measured to be approximately 30~45 nm. The results revealed that some aggregation of TiO_2_ grains occurs during laser-liquid interaction if we compare the particle size from SEM and from the XRD data calculation.

**Figure 4.**
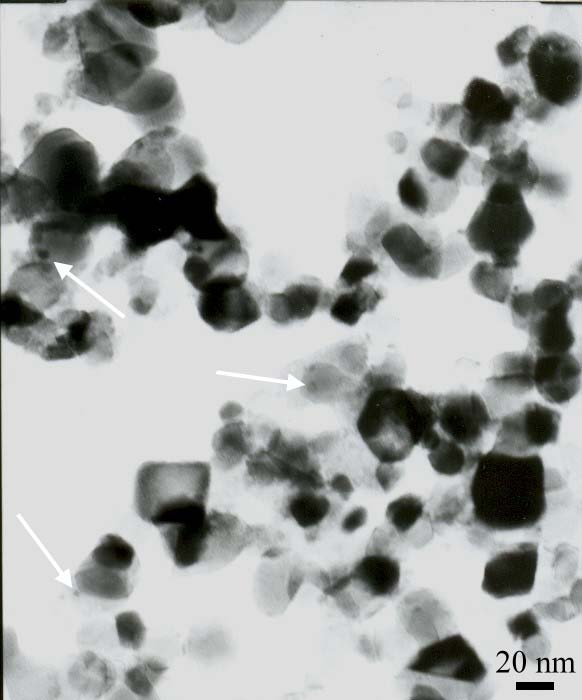
TEM image of 2.0 wt% Ag in TiO_2_ nanoparticles. Some of Ag nanoparticles were indicated by the arrows.

Figure 4 shows the TEM image of 2.0 wt% Ag/TiO_2_ nanoparticles. Small spherical Ag nanoparticles (some of these particles are indicated by arrows) were observed scattered on the surface of TiO_2_. For Ag particles, the diameter ranges from 3 to 6 nm, and the diameter of TiO_2_ was found to be in the scope of 20~40 nm. It suggests that small silver nanoparticles can be prepared by pulsed-laser irradiation applied to AgNO_3_ and TiO_2_ system, and that a good dispersion of these particles on the surface of the matrix is probable. The average diameter of particles from TEM image was found to be in accordance with the results of the SEM image.

**Figure 5.**
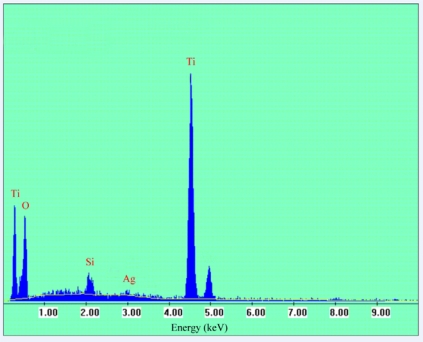
EDX diagram of 2.0 wt% Ag/TiO_2_ catalyst.

We have also performed the EDX analysis on the 2.0 wt% Ag/TiO_2_ catalyst. The EDX diagram of 2.0 wt% Ag/TiO_2_ is shown in Figure 5, where the silver signals are found at around 3.00 keV [25]. Though the peaks of silver are insignificant due to its content in TiO_2_ matrix, it can be indicative of the presence of Ag particles in catalyst.

### 2.3. Photodegradation of Methylene Blue by Ag/TiO_2_

**Figure 6.**
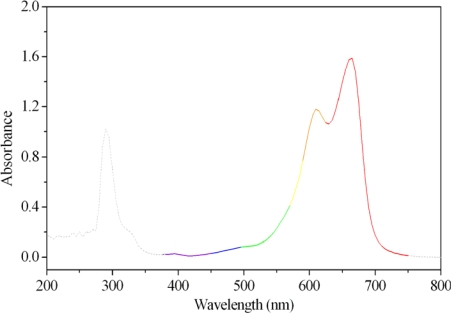
UV-Vis spectrum of methylene blue solution, with maximum absorption at 668 nm.

In this article, methylene blue was used as a model pollutant for evaluation of the photocatalytical efficiency of the laser-induced Ag/TiO_2_ nanoparticles. Methylene blue, with an absorption maximum at 668 nm in visible light region, as shown in Figure 6, is usually used in mixed indicators or as a redox indicator. Hence, the amount of MB was measured quantitatively with the absorption of light at 668 nm.

**Figure 7.**
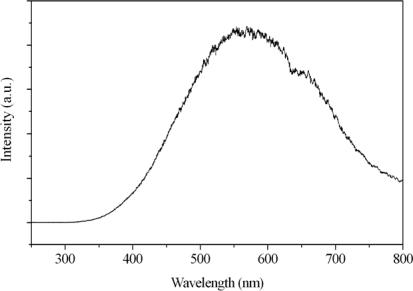
Light emitting spectrum of the halogen lamp used in degradation procedure.

Figure 7 presents the light emitting spectrum of the halogen lamp applied in the degradation procedure, its emitting wavelength ranges from 350 nm to 800 nm with the predominant peak at 575 nm. The power of the halogen lamp is 150 W. Because the band gap of TiO_2_ is 3.0~3.2 eV, a wavelength greater than 387~414 nm will be short of any absorption by TiO_2_-only, as shown in Figure 1. If the photocatalysis of Ag/TiO_2_ in visible-light range improves through halogen lamp irradiation, it can be attributed to the absorption of visible light by Ag particles doped in the TiO_2_.

**Figure 8.**
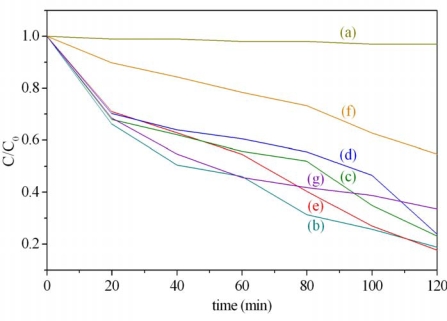
Photodegradation of methylene blue solution by using halogen lamp irradiation with (a) no catalyst, (b) 0.0, (c) 0.5, (d) 1.0, (e) 2.0, (f) 5.0, and (g) 10.0 wt% of Ag in TiO_2_.

Before the photodegradation experiments were conducted, the possible reactions such as adsorption of MB by nanoparticles and direct light-degradation were studied as follows: the adsorption of MB by Ag/TiO_2_ was carried out by putting the photocatalyst into the MB solution, an aliquot of solution was examined successively by UV-Vis at 20-min intervals. From our experiments, it took about 20 min. to reach the saturated adsorption of MB by Ag/TiO_2_; the direct light-degradation test was carried out by illuminating the solution directly with a halogen lamp without photocatalysts. There is no obvious degradation of MB in solution in two hours, just as the result observed in Figure 8(a). These effects were taken into consideration for the photodegradation experiments of this work.

The pH value of the solution also plays an important role in the photodegradation process; it was found that the maximum rate of photocatalytic degradation by TiO_2_ is achieved at pH 6.9 [26]. Hence, the pH condition of photodegradation experiments of this work was controlled before the degradation process for optimum results. Figure 8 shows the photodegradation of MB solution in the presence of Ag/TiO_2_ initiated by the illumination of a halogen lamp. Line (a) in Figure 8 presents the degradation reaction of MB in the absence of catalyst, revealing no change in MB concentration under this condition. Rapid conversion of MB (about 28~35%) is found for the first 20 min. irradiation except line (f), which is about 10% conversion for 5.0 wt% Ag/TiO_2_. The conversion of MB progressed gradually afterward, reaching 54, 45, 40, 45, 22, and 55% for 0.0, 0.5, 1.0, 2.0, 5.0, and 10.0 wt% Ag/TiO_2_ at 1 h irradiation, respectively. The maximum efficiency was achieved by 2.0 wt% Ag/TiO_2_ for an 82.3% conversion at 2 h irradiation. The degradation of MB displayed a first-order reaction, rate constants of various ratios of Ag/TiO_2_ are listed in Table 1. However, zero order of the degradation reaction for higher concentrations was reported elsewhere [5].

In our photodegradation tests, the most efficient photocatalyt of all the ratios of Ag to TiO_2_ tested was found to be the 2.0 wt% Ag/TiO_2_. A maximum of 82.3% MB degradation by Ag/TiO_2_ in aqueous solution was achieved. However, the photodegradation efficiency of Ag/TiO_2_ did not exhibit the increasing trend with Ag content greater than 2.0 wt%, the efficiency decreases with higher Ag contents were observed instead. According to other studies [23], the detrimental effects of Ag in greater amount relative to TiO_2_ can be explained as follows: (1) the increasing amount of Ag on TiO_2_ becomes a barrier hindering the contact of dye molecules with TiO_2_; (2) the Ag particles prevent the light absorption of TiO_2_; (3) excess Ag on TiO_2_ facilitates the recombination of electron-hole pairs of TiO_2_ in bulk or at the surface; (4) greater contents of Ag particles inhibit the reaction of oxygen in the photocatalytic process. In view of this, it is important to adjust the Ag and TiO_2_ composition to the proper ratio in order to acquire the optimum photodegradation efficiency of Ag/TiO_2_ photocatalyst dealing with MB aqueous solution.

## 3. Experimental

### 3.1. Materials

P25 TiO_2_ was purchased from Degussa (Germany). Silver nitrate was purchased from Hayashi Pure Chemical Industry (99.5% purity). Isopropyl alcohol was purchased from Hayashi Pure Chemical Industry (99.9% purity). Methylene Blue was purchased from Panreac Quimica SA. Deionized water was used throughout all experiments. All reagents were used without further purification.

### 3.2. Preparation of Ag/TiO_2_ Photocatalysts

Silver nitrate was used as Ag precursor. The various ratios of AgNO_3_ to TiO_2_ were prepared by mixing 0.5, 1.0, 2.0, 5.0, and 10.0 weight percents of AgNO_3_ in TiO_2_, with total weight maintained at 1.0 g. Then 10 g of isopropanol was added to each mixture. Each solution was sonicated 30 min. for uniform mixing. A pulsed-laser (25 mJ, 532 nm, 10 Hz Nd:YAG) light was applied from top of the container to each sample for 60 min. Finally, the air-dry samples were annealed at 200 °C for 60 min.

### 3.3. Characterization of the Prepared Catalysts

The UV-Vis spectra of Ag/TiO_2_ were carried out on a Hitachi U-3010 spectrophotometer with an integrated sphere, wavelength from 200 nm through 800 nm, with a scanning rate of 300 nm/min. The XRD analyses were carried out on a Shimadzu XD-D1 X-ray diffractometer, using Cu-Kα radiation with λ = 0.154051 nm, in a range of 20–80° (2θ). TEM images were recorded on a JEOL JEM 1200-EX electron microscope. SEM images were recorded on a Philips XL40 microscope.

### 3.4. Determination of Photocatalytic Activities of Ag/TiO_2_

The installation for photocatalytic degradation of MB by Ag/TiO_2_ was assembled as the following: A 150 W halogen lamp, wavelength range from 350 nm to 800 nm with the predominant peak at 575 nm, was used as the light source placed on top of the setup. 1.0 g of Ag/TiO_2_ was added in 100 mL of MB (7,000 mg/L) solution. After the mixture was sonicated for 30 min., the halogen lamp was turned on to initiate the reaction. During the irradiation periods, 5 mL of solution were taken out of the reactor and centrifuged to separate the solid from the solution at 20-min intervals. An UV-Vis spectroscopy was used to detect the MB concentration of each centrifuged solution, which was collected at 20-min interval for two hours of reaction time in all.

## 4. Conclusions

We have introduced a laser-induced method of doping Ag nanoparticles onto a TiO_2_ matrix, with laser-liquid interaction of AgNO_3_ and TiO_2_ in a liquid medium of isopropanol. Apart from other methods of preparation of Ag modified TiO_2_ such as photoreduction, chemical reduction, and sol-gel process, the method we proposed in this article provides a simple, straightforward way for enhancing its photocatalytic efficiency. XRD, TEM and SEM results of Ag/TiO_2_ indicated that narrow size distributions of Ag nanoparticles on TiO_2_ were achieved by the laser-induced method. The Ag nanoparticles deposited on TiO_2_ act like the electron traps of the matrix, preventing recombination of electron-hole pairs on the surface of TiO_2_ and improving charge transfer processes. The photocatalysis efficiency of Ag doped TiO_2_ was tested by the degradation of MB in aqueous solution. A maximum of 82.3% MB degradation under 2 h of halogen lamp illumination using 2 wt% Ag/TiO_2_, prepared by the laser-induced method of this article, is observed.

## Figures and Tables

**Table 1.** Photocatalytic efficiencies for photodegradation of MB by various ratios of Ag/TiO_2_.

**wt% Ag/TiO_2_**	**Conversion after 2 h (%)**	**Rate constant (min^−1^)**
0.5	76.9	1.1 × 10^−2^
1.0	76.2	9.4 × 10^−3^
2.0	82.3	1.4 × 10^−2^
5.0	45.4	4.8 × 10^−3^
10.0	66.5	8.4 × 10^−3^
